# Non-diabetic Kidney Disease in Type 2 Diabetes: From Kidney Biopsy to Precision Medicine

**DOI:** 10.7759/cureus.100716

**Published:** 2026-01-03

**Authors:** Sreenath Sreedharan, Mohandas M. K., Vismaya K. B., Nikhil Raju

**Affiliations:** 1 Nephrology, Government Medical College, Thiruvananthapuram, IND; 2 Urology, Government Medical College, Thiruvananthapuram, IND

**Keywords:** diabetic kidney disease, glomerulonephritis, non-diabetic kidney disease, renal biopsy, type 2 diabetes mellitus

## Abstract

Non-diabetic kidney disease (NDKD) is increasingly recognized as a significant and often underappreciated problem in people with type 2 diabetes mellitus (T2DM), accounting for a substantial proportion of kidney abnormalities identified through biopsy. Recent evidence shows that many patients do not have purely diabetic kidney changes; instead, a large share have non-diabetic or mixed pathologies. These findings challenge the long-standing assumption that kidney disease in diabetes is always due to diabetic nephropathy.

This narrative review synthesizes current evidence on the prevalence, clinical predictors, diagnostic tools, and therapeutic strategies for NDKD in T2DM, with a focus on combining renoprotective therapy with disease-specific immunomodulation.

We reviewed PubMed, EMBASE, and Scopus (January 2020-December 2024), including systematic reviews, meta-analyses, randomized controlled trials, cohort studies, and clinical guidelines, addressing NDKD in diabetes.

NDKD shows substantial geographic variation. IgA nephropathy (IgAN) is most common in Asian cohorts (25%-43%), whereas membranous nephropathy (MN, 20%-30%) and focal segmental glomerulosclerosis (FSGS, 18%-25%) predominate in Western populations. Key clinical predictors include the absence of diabetic retinopathy (odds ratio (OR) 0.15, 95% confidence interval (CI) 0.09-0.26 for DKD), diabetes duration under five years (OR 5.8, 95% CI 3.2-10.5 for NDKD), hematuria (OR 7.2, 95% CI 4.1-12.6), and rapid estimated glomerular filtration rate (eGFR) decline greater than 5 mL/min/1.73 m² per year (OR 4.3, 95% CI 2.8-6.7). Prediction models report areas under the curve (AUC) of 0.82-0.89. Emerging tools, including urinary proteomic panels (AUC 0.88-0.91) and artificial intelligence (AI)-assisted histopathology (AUC 0.91), are promising but do not replace kidney biopsy. Renoprotective therapies remain essential. Sodium-glucose cotransporter 2 inhibitors (SGLT2i) show hazard ratios (HR) of 0.61-0.72 for kidney outcomes; glucagon-like peptide-1 receptor agonists (GLP-1 RA) around 0.76; and mineralocorticoid receptor antagonists (MRA) about 0.82. Disease-specific immunomodulation is equally important, including rituximab for MN (HR 3.82, 95% CI 2.42-6.02) and complement-targeting therapies for IgAN.

NDKD requires integrated management that combines standard renoprotection (SGLT2i, renin-angiotensin system (RAS) blockade, GLP-1 RA, MRA) with histology-guided immunosuppression. Kidney biopsy should be pursued when results are expected to change management decisions.

Overall, NDKD affects one-third to one-half of patients with T2DM and kidney disease. Early recognition, maintaining clinical suspicion in patients with atypical features, appropriate biopsy use, and tailored treatment strategies are essential to improving outcomes.

## Introduction and background

Chronic kidney disease (CKD) has become a major global public health concern, with the Global Burden of Disease Study estimating that 697.5 million people were affected in 2017, a 29.3% increase since 1990 [1]. In individuals with type 2 diabetes mellitus (T2DM), kidney disease remains one of the most common and serious microvascular complications, and diabetic kidney disease (DKD) is now the leading cause of end-stage kidney disease (ESKD) in most developed countries [2]. Traditionally, kidney disease in diabetes has been attributed primarily to DKD, which is defined by the classic histopathologic features of glomerular basement membrane thickening, mesangial expansion, and nodular glomerulosclerosis (Kimmelstiel-Wilson lesions) [3].

However, growing evidence from kidney biopsy registries across several regions challenges this assumption. Studies now show that non-diabetic kidney disease (NDKD), which includes a wide range of glomerular, tubular, vascular, and interstitial disorders, accounts for a substantial proportion of kidney dysfunction in people with diabetes [2,4-6]. A landmark meta-analysis by Fiorentino et al. that reviewed 48 biopsy studies involving 4,876 diabetic patients found that isolated DKD was present in only 37% (range 25%-52%) of cases. In contrast, pure NDKD accounted for 36% (range 28%-48%), while mixed lesions (both DKD and NDKD) represented 27% (range 18%-35%) of biopsy diagnoses [2]. These findings have been consistently confirmed in recent multicenter biopsy cohorts from Asia, Europe, and North America [4,5,7].

The clinical importance of distinguishing NDKD from DKD goes beyond academic classification, as it directly influences treatment decisions. While DKD management focuses on glycemic control, blood pressure management, renin-angiotensin system (RAS) blockade, and newer renoprotective agents such as SGLT2 inhibitors and GLP-1 receptor agonists, many forms of NDKD require disease-specific immunomodulatory or targeted therapies that can substantially change patient outcomes [6,8,9]. For example, membranous nephropathy (MN) in a patient with diabetes may achieve complete remission with rituximab [10], IgA nephropathy (IgAN) may respond to complement-directed therapy [11], and focal segmental glomerulosclerosis (FSGS) may require corticosteroids or calcineurin inhibitors [12]. Failure to identify NDKD can therefore lead to missed opportunities for effective treatment and potentially preventable progression to ESKD [13].

The heterogeneity of NDKD creates diagnostic challenges, as clinical presentations can overlap with those of DKD. Although several clinical predictors, such as absence of diabetic retinopathy, shorter duration of diabetes before onset of kidney disease, microscopic hematuria, and rapid decline in eGFR, have been associated with a higher likelihood of NDKD, no individual feature or combination of features offers sufficient sensitivity or specificity to reliably confirm or exclude NDKD without a kidney biopsy [14,15]. This diagnostic gap has led to increasing interest in non-invasive tools, including serum and urinary biomarkers, urinary proteomic panels, and artificial intelligence (AI)-assisted diagnostic models, although none have yet demonstrated accuracy high enough to replace histopathologic assessment [16-18].

Parallel advances in therapeutic nephrology have added further complexity to the management of diabetic patients with kidney disease. Landmark clinical trials have shown that SGLT2 inhibitors offer strong kidney protection across a wide range of CKD causes, including both DKD and NDKD, with benefits also seen in individuals without diabetes [6,19,20]. Likewise, nonsteroidal mineralocorticoid receptor antagonists (MRAs) such as finerenone have demonstrated kidney and cardiovascular benefits in diabetic CKD [21,22]. As a result, these broader “disease-agnostic” renoprotective therapies must now be combined with “disease-specific” immunomodulatory treatments for NDKD. This shift requires moving away from traditional, siloed management and toward integrated, multifaceted therapeutic strategies [23].

The geographic distribution of NDKD also varies considerably, reflecting both true epidemiologic differences and potentially differing biopsy practices across regions. In many Asian countries, IgA nephropathy (IgAN) is the leading NDKD diagnosis, representing 25%-43% of cases in biopsy series from China, Japan, Korea, and India [4,5,24]. In contrast, biopsy registries in Western nations more commonly report membranous nephropathy (MN) and focal segmental glomerulosclerosis (FSGS), each comprising roughly 18%-30% of NDKD diagnoses [2,25]. Recognizing these regional patterns can help clinicians maintain appropriate diagnostic suspicion and ensure timely kidney biopsy when warranted [26].

This narrative review summarizes current evidence on the epidemiology, clinical predictors, diagnostic modalities, and therapeutic approaches for NDKD in patients with T2DM. We describe the spectrum of NDKD entities, outline clinical and laboratory clues that may help differentiate NDKD from DKD, review emerging non-invasive diagnostic tools, and propose an integrated management model that pairs disease-agnostic renoprotective therapies with disease-specific immunomodulation. Ultimately, we highlight the importance of maintaining a high index of suspicion for NDKD in diabetic patients with kidney disease, using kidney biopsy when results are likely to change management, and implementing individualized treatment strategies based on histopathologic findings [27].

Methods

Study Design and Search Strategy

This narrative review was conducted to summarize current evidence on NDKD in patients with T2DM. We searched PubMed, EMBASE, and Scopus from January 2020 to December 2024 and included key landmark studies published before 2020 that contributed foundational knowledge in this area. Search terms combined “non-diabetic kidney disease”, “diabetic kidney disease”, “type 2 diabetes”, “kidney biopsy”, “IgA nephropathy”, “membranous nephropathy”, “FSGS”, “glomerulonephritis”, “SGLT2 inhibitors”, and “immunomodulation” and related Medical Subject Headings (MeSH) terms.

Inclusion and Exclusion Criteria

We included systematic reviews, meta-analyses, randomized controlled trials, cohort studies, diagnostic accuracy studies, kidney biopsy registry data, and clinical practice guidelines that examined the prevalence, diagnosis, or management of NDKD in diabetic populations. Studies were prioritized when they had clearly defined populations, robust methodology, and reliable diagnostic criteria. Studies focusing only on type 1 diabetes were excluded unless the findings were applicable to T2DM. Case reports and small case series (fewer than 10 patients) were excluded unless they introduced novel diagnostic or therapeutic insights.

Data Extraction and Synthesis

Because this is a narrative review, we did not conduct formal systematic data extraction or use structured risk-of-bias tools (e.g., Cochrane Risk of Bias, QUADAS-2). Instead, we used a thematic synthesis approach. Evidence was organized into major clinical domains: epidemiology and prevalence, histopathologic patterns, clinical predictors and risk stratification, advances in diagnostics (including biomarkers and AI), renoprotective therapies, disease-specific immunomodulation, and indications for kidney biopsy. Quantitative data such as prevalence estimates, odds ratios, hazard ratios, AUC values, and treatment effect sizes were extracted when available, along with confidence intervals and p-values.

Quality Assessment

Although we did not perform a formal risk-of-bias assessment, we gave greater weight to randomized controlled trials, large multicenter cohorts, and meta-analyses published in reputable peer-reviewed journals. We also considered study limitations such as selection bias in biopsy cohorts, heterogeneity in diagnostic criteria, and the limited external validation of prediction models and biomarker panels. Evidence from major clinical practice guidelines, including KDIGO recommendations, was used to contextualize findings within current standards of care [27,28].

Limitations of Narrative Review Design

This narrative review has inherent limitations compared with systematic reviews or meta-analyses. The search strategy, while comprehensive, was not exhaustive, and dual independent screening and data extraction were not performed. The synthesis reflects expert interpretation of available evidence rather than quantitative pooling of results. In addition, publication bias and selective reporting could not be formally evaluated. Nonetheless, the narrative format allows for integration of diverse study designs and supports discussion of complex, evolving clinical issues, an approach that is well suited to the multifaceted nature of NDKD in diabetic patients.

## Review

Evidence synthesis and results

Prevalence and Geographic Distribution of NDKD

The prevalence of NDKD among diabetic patients with kidney disease varies substantially, depending on whether estimates are derived from kidney biopsy series or population-based studies. Biopsy-based estimates inherently reflect selection bias, as kidney biopsy is typically pursued in diabetic patients with atypical features suggestive of NDKD or when biopsy findings would potentially alter management [26,29]. Nonetheless, biopsy registries provide the only definitive data on histopathologic diagnoses and serve as the foundation for understanding NDKD epidemiology [2].

The seminal meta-analysis by Fiorentino et al. pooled data from 48 kidney biopsy studies encompassing 4,876 diabetic patients across multiple countries and continents [2]. This analysis revealed that isolated DKD was present in 37% of biopsied diabetic patients (range 25%-52% across studies), pure NDKD without any DKD features was found in 36% (range 28%-48%), and mixed pathology with concurrent DKD and NDKD was identified in 27% (range 18%-35%) [2]. These findings established that NDKD, either alone or in combination with DKD, accounts for the majority (63%) of kidney disease in diabetic patients undergoing biopsy, a proportion far higher than traditionally assumed [2,30].

More recent multicenter biopsy series have validated and refined these prevalence estimates. A 2024 Chinese multicenter study of 235 diabetic patients with kidney disease found pure DKD in 34%, pure NDKD in 43%, and mixed pathology in 23% of biopsied cases [4]. An Indian single-center prospective study of 186 consecutive diabetic patients with kidney disease reported pure DKD in 28%, pure NDKD in 48%, and mixed pathology in 24% [5]. A European registry analysis identified NDKD in 39% of diabetic patients undergoing kidney biopsy [7]. Collectively, contemporary studies consistently demonstrate that NDKD accounts for 30%-50% of kidney disease in biopsied diabetic populations, with combined NDKD and mixed pathology affecting the majority of patients in many series [4,5,7].

Geographic and Histopathologic Heterogeneity

The histopathologic spectrum of NDKD demonstrates marked geographic variation, reflecting both genuine epidemiologic differences in glomerular disease prevalence and potentially divergent clinical practices regarding biopsy indications [26,29]. In Asian populations, IgAN emerges as the predominant NDKD entity, accounting for 25%-43% of all NDKD diagnoses in biopsy series from China, Japan, Korea, and India [4,5,24,31]. This predominance likely reflects the high background prevalence of IgAN in Asian populations generally, where IgAN is the most common primary glomerular disease even among non-diabetic individuals [32,33].

In contrast, Western biopsy registries from North America and Europe identify MN as the most common NDKD diagnosis, accounting for 20%-30% of NDKD cases, followed closely by FSGS (18%-25%) and acute or chronic tubulointerstitial nephritis (10%-18%) [2,25,34]. Minimal change disease, while less common, accounts for 8%-12% of NDKD in Western series and often presents with sudden-onset nephrotic syndrome in diabetic patients [34,35].

Indian biopsy series demonstrate a unique distribution pattern, with a more balanced distribution across multiple NDKD entities: MN (14%-18%), FSGS (12%-17%), IgAN (15-20%), and acute tubulointerstitial nephritis (10%-15%) [5]. This heterogeneity may reflect India's geographic and ethnic diversity, as well as high rates of nephrotoxic medication exposure and chronic infections that predispose to tubulointerstitial disease [5,36].

Clinical Predictors and Risk Stratification for NDKD

Given the therapeutic and prognostic implications of distinguishing NDKD from DKD, substantial research has focused on identifying clinical and laboratory features that predict the presence of NDKD in diabetic patients with kidney disease. While no single feature possesses sufficient diagnostic accuracy to definitively confirm or exclude NDKD, several clinical predictors have demonstrated robust associations with NDKD in large cohort studies and meta-analyses [14,15,37].

Absence of Diabetic Retinopathy

The absence of diabetic retinopathy in a diabetic patient with kidney disease is the single most powerful clinical predictor of NDKD, with meta-analyses demonstrating an OR of approximately 0.15 (95% CI 0.09-0.26) for DKD when retinopathy is absent, conversely indicating substantially elevated odds for NDKD [14,15]. This association reflects the parallel microvascular pathology affecting retinal and glomerular capillaries in diabetic microangiopathy, such that the presence of DKD is typically (though not invariably) accompanied by retinopathy [38,39].

However, several important caveats must be recognized. First, the correlation between retinopathy and DKD is stronger in type 1 diabetes than in T2DM, likely due to the more homogeneous natural history and clearer temporal relationship between hyperglycemia and microvascular complications in type 1 diabetes [40]. In T2DM, the longer asymptomatic period before diagnosis means that some patients may have had unrecognized hyperglycemia for years before diabetes diagnosis, potentially allowing time for the development of DKD before retinopathy manifests [41]. Second, advanced DKD (particularly nodular glomerulosclerosis) can occasionally occur without retinopathy in approximately 10%-15% of cases, particularly in T2DM [42].

Despite these limitations, the absence of diabetic retinopathy should prompt heightened suspicion for NDKD and consideration of kidney biopsy, particularly when other atypical features are present [15,26].

Diabetes Duration and Temporal Relationships

The natural history of DKD typically involves a latency period of 10-15 years from diabetes diagnosis to clinically evident nephropathy, reflecting the cumulative effect of chronic hyperglycemia and metabolic stress on glomerular structure and function [43,44]. Consequently, the development of significant kidney disease within five years of diabetes diagnosis is atypical for DKD and strongly suggests alternative diagnoses [14,15].

Meta-analyses have demonstrated that diabetes duration less than five years at the time of kidney disease onset is associated with an OR of approximately 5.8 (95% CI 3.2-10.5) for NDKD [15]. This relationship is particularly robust in type 1 diabetes, where the onset of diabetes is typically well-defined and the natural history more predictable [45].

Microscopic Hematuria and Active Urinary Sediment

Microscopic hematuria, defined as more than five red blood cells per high-power field on urine microscopy, is uncommon in DKD and suggests glomerular inflammation characteristic of proliferative or necrotizing glomerulonephritides [46,47]. Meta-analyses demonstrate that hematuria is associated with an OR of approximately 7.2 (95% CI 4.1-12.6) for NDKD [15]. Particularly suggestive of glomerular disease are dysmorphic red blood cells (RBCs that have been distorted by passage through damaged glomerular capillaries) and red blood cell casts (RBCs embedded in Tamm-Horsfall protein matrix, indicating glomerular origin and tubular transit) [48,49].

Active urinary sediment, characterized by the presence of RBC casts, white blood cell casts, dysmorphic RBCs, or significant proteinuria with cellular elements, demonstrates an OR of approximately 6.8 (95% CI 3.9-11.8) for NDKD [15].

Rapid eGFR Decline

While DKD typically follows a gradual progressive course with annual eGFR decline of 2-4 mL/min/1.73 m² per year in patients with established albuminuric DKD, more rapid kidney function decline suggests alternative diagnoses or superimposed acute kidney injury [50,51]. Rapid eGFR decline, defined as loss exceeding 5 mL/min/1.73 m² per year, is associated with an OR of approximately 4.3 (95% CI 2.8-6.7) for NDKD [15].

Proteinuria Pattern and Magnitude

The pattern and magnitude of proteinuria provide important diagnostic clues. Nephrotic-range proteinuria (>3.5 g/day or UACR >3,500 mg/g) occurring in the setting of preserved kidney function (eGFR > 60 mL/min/1.73 m²) is atypical for early DKD and strongly suggests primary glomerular disease, particularly MN, minimal change disease, or FSGS [15,26,52]. Meta-analyses demonstrate an OR of approximately 3.2 (95% CI 1.8-5.7) for NDKD in this clinical scenario [15].

Multivariate Prediction Models

Recognizing that individual clinical features have limited positive predictive value when considered in isolation, several research groups have developed multivariate prediction models combining multiple clinical and laboratory variables to improve diagnostic accuracy for NDKD [14,15,53].

The Chinese Five-Variable Model, derived from a cohort of 620 diabetic patients undergoing kidney biopsy and validated in an external cohort of 227 patients, incorporates diabetes duration, presence or absence of diabetic retinopathy, urinary RBC count, serum albumin, and hemoglobin A1c [14]. This model achieved an area under the receiver operating characteristic curve (AUC) of 0.89 in the derivation cohort and 0.82 in external validation, substantially outperforming individual clinical predictors [14].

An Indian point-based prediction score incorporating six weighted clinical variables (retinopathy status, diabetes duration, hematuria, rate of eGFR decline, proteinuria magnitude, and urinary sediment) achieved an AUC of 0.84 in a prospective cohort of 186 diabetic patients [5]. The score ranges from 0 to 12 points, with a high-risk stratum (score ≥8) identifying a population with 82% prevalence of NDKD, while a low-risk stratum (score ≤3) had only 12% prevalence of NDKD [5].

Diagnostic Advances: Biomarkers and AI

The invasive nature of kidney biopsy and its associated risks have motivated the search for non-invasive diagnostic tools to differentiate DKD from NDKD [54,55]. While kidney biopsy remains the reference standard for definitive diagnosis, emerging biomarkers and AI applications show promise for risk stratification, selection of biopsy candidates, and potentially monitoring disease activity and treatment response [16,17,56].

Urinary Biomarkers and Proteomic Profiling

Urinary biomarkers offer the advantage of non-invasive sampling with direct access to kidney-derived proteins and metabolites [57]. Individual biomarkers reflecting specific aspects of kidney injury have been investigated as potential diagnostic tools [58-60].

Multi-marker proteomic panels have shown more promising diagnostic performance by capturing multiple pathophysiologic pathways simultaneously [61-63]. Urinary proteomic panels have achieved AUCs of 0.88-0.91 for distinguishing DKD from NDKD in multiple validation cohorts, with a sensitivity of approximately 84% and a specificity of 87% [61,64].

Despite these promising results, clinical implementation of proteomic classifiers has been limited by several challenges, including the need for specialized equipment, standardization issues, and cost considerations [65,66].

AI and Machine Learning

AI applications in nephropathology represent a transformative frontier with potential to enhance diagnostic accuracy, reproducibility, and efficiency of histopathologic evaluation [67-69]. Deep learning algorithms for automated classification of glomerular diseases from digitized kidney biopsies have achieved an AUC of 0.91 for multi-class classification of glomerular diseases, successfully differentiating DKD, IgA nephropathy, MN, FSGS, and minimal change disease [67].

AI algorithms utilizing transmission electron microscopy have achieved approximately 88% accuracy in distinguishing primary from secondary FSGS based on ultrastructural features [70].

Table 1 summarizes the trials addressing diabetic and non-diabetic CKD.

**Table 1:** Major renoprotective trials in diabetic and non-diabetic CKD SGLT2i: sodium-glucose cotransporter-2 inhibitor, MRA: mineralocorticoid receptor antagonist, T2DM: type 2 diabetes mellitus, CKD: chronic kidney disease, ESKD: end-stage kidney disease, Cr: creatinine, eGFR: estimated glomerular filtration rate, CV: cardiovascular, HR: hazard ratio, CI: confidence interval, RRR: relative risk reduction, RAAS: renin-angiotensin-aldosterone system, NS: not significant.

Trial	Drug class	Population	N	Primary kidney outcome	HR (95% CI)	p-value	Key findings
CREDENCE [20]	SGLT2i (Canagliflozin)	T2DM + CKD + albuminuria	4,401	ESKD, Cr doubling, kidney death	0.70 (0.59-0.82)	<0.001	30% RRR; benefit independent of baseline RAAS blockade
DAPA-CKD [6]	SGLT2i (Dapagliflozin)	CKD ± diabetes	4,304	ESKD, ≥50% eGFR decline, kidney death	0.61 (0.51-0.72)	<0.001	39% RRR; consistent benefit in diabetic and non-diabetic CKD
EMPA-KIDNEY [19]	SGLT2i (Empagliflozin)	CKD ± diabetes (broad spectrum)	6,609	Kidney disease progression or CV death	0.72 (0.64-0.82)	<0.001	28% RRR; benefit in eGFR as low as 20 mL/min/1.73 m²
FIDELIO-DKD [21]	MRA (Finerenone)	T2DM + CKD + albuminuria	5,674	Kidney failure, ≥40% eGFR decline, kidney death	0.82 (0.73-0.93)	0.001	18% RRR; additive to optimized RAAS inhibition
FIGARO-DKD [22]	MRA (Finerenone)	T2DM + CKD (earlier stages)	7,437	Kidney outcomes (secondary endpoint)	0.87 (0.76-1.01)	NS	13% RRR for kidney outcomes; primary endpoint was CV

Therapeutic Integration: Disease-Agnostic Renoprotection

The past decade has witnessed a revolution in therapeutic nephrology, with multiple landmark clinical trials demonstrating robust kidney protection with novel agents that benefit both diabetic and non-diabetic CKD across diverse etiologies [6,19,20,71]. These "disease-agnostic" renoprotective therapies must now be integrated with "disease-specific" immunomodulatory interventions for NDKD [23,72].

Sodium-Glucose Cotransporter-2 Inhibitors

SGLT2i have emerged as foundational renoprotective therapies with benefits extending far beyond glycemic control [73,74]. The CREDENCE trial demonstrated a 30% relative risk reduction (hazard ratio 0.70, 95% CI 0.59-0.82) for the primary composite kidney outcome [20]. The DAPA-CKD trial extended these findings to a broader CKD population, with dapagliflozin reducing the primary composite kidney outcome by 39% (hazard ratio 0.61, 95% CI 0.51-0.72), with consistent benefit in diabetic and non-diabetic participants [6]. The EMPA-KIDNEY trial further confirmed broad renoprotective effects with empagliflozin, reducing outcomes by 28% (hazard ratio 0.72, 95% CI 0.64-0.82) [19].

Glucagon-Like Peptide-1 Receptor Agonists

GLP-1 receptor agonists have demonstrated kidney benefits beyond glycemic control [75,76]. Meta-analyses of cardiovascular outcomes trials have demonstrated that GLP-1 RAs reduce composite kidney outcomes by approximately 20%-24%, corresponding to hazard ratios of 0.76-0.80 [75,77,78].

Mineralocorticoid Receptor Antagonists: Finerenone

Finerenone, a novel nonsteroidal MRA, was specifically developed to target cardiorenal inflammation and fibrosis while minimizing hyperkalemia risk [79,80]. The FIDELIO-DKD trial demonstrated that finerenone reduced the primary composite kidney outcome by 18% (hazard ratio 0.82, 95% CI 0.73-0.93) [21]. The FIGARO-DKD trial demonstrated a 13% relative risk reduction for kidney outcomes (hazard ratio 0.87, 95% CI 0.76-1.01) [22].

Renin-Angiotensin System Inhibition

RAS inhibition with angiotensin-converting enzyme inhibitors (ACEi) or angiotensin receptor blockers (ARB) remains foundational therapy for proteinuric CKD across DKD and NDKD [81,82]. Landmark trials established that RAS blockade reduces proteinuria, slows eGFR decline, and delays progression to ESKD [83,84,85].

Table 2 provides the clinical predictors of NDKD in diabetic patients.

**Table 2:** Clinical predictors of NDKD in diabetic patients NDKD: non-diabetic kidney disease, DKD: diabetic kidney disease, OR: odds ratio, CI: confidence interval, RBC: red blood cell, HPF: high-power field, eGFR: estimated glomerular filtration rate, UACR: urinary albumin-to-creatinine ratio, MN: membranous nephropathy, MCD: minimal change disease, FSGS: focal segmental glomerulosclerosis, DR: diabetic retinopathy, HbA1c: hemoglobin A1c, AUC: area under the curve. *Note: OR < 1.0 indicates reduced odds of DKD (i.e., increased odds of NDKD when retinopathy is absent).

Clinical predictor	Odds ratio (95% CI)	Sensitivity	Specificity	Key studies	Clinical interpretation
Absence of diabetic retinopathy	OR 0.15 (0.09-0.26) for DKD*	74%	88%	Liang et al. [14]; Mou et al. [15]	Most powerful single predictor; retinopathy absence strongly suggests NDKD
Diabetes duration <5 years	OR 5.8 (3.2-10.5) for NDKD	62%	81%	Liang et al. [14]; Mou et al. [15]	DKD typically requires >10 years; early kidney disease suggests alternative diagnosis
Microscopic hematuria (>5 RBC/HPF)	OR 7.2 (4.1-12.6) for NDKD	58%	86%	Liang et al. [14]; Mou et al. [15]	Uncommon in DKD; suggests glomerular inflammation
Rapid eGFR decline (>5 mL/min/1.73 m²/year)	OR 4.3 (2.8-6.7) for NDKD	48%	83%	Mou et al. [15]; Cattran et al. [50]	DKD typically declines 2-4 mL/min/1.73 m²/year; rapid decline suggests NDKD
Nephrotic proteinuria (UACR >3,500 mg/g) with preserved eGFR (>60)	OR 3.2 (1.8-5.7) for NDKD	42%	79%	Mou et al. [15]; Kidney Disease: Improving Global Outcomes (KDIGO) CKD Work Group [26]	Suggests primary glomerular disease (MN, MCD, FSGS)
Active urinary sediment (RBC casts, dysmorphic RBCs)	OR 6.8 (3.9-11.8) for NDKD	51%	91%	Mou et al. [15]; Fogazzi et al. [46]	Highly specific for glomerulonephritis
Multivariate prediction models	AUC	Sensitivity	Specificity	Components	Validation Status
Chinese Five-Variable Model	0.89 (derivation) 0.82 (validation)	84%	78%	Diabetes duration, DR, RBC count, albumin, HbA1c	External validation completed [14]
Indian point-based score (0-12 points)	0.84	79%	81%	DR, duration, hematuria, eGFR slope, proteinuria, sediment	Single-center validation [5]

Disease-Specific Immunomodulation for NDKD

While disease-agnostic renoprotective therapies benefit both DKD and NDKD, specific glomerular diseases require targeted immunomodulatory or disease-specific interventions that can fundamentally alter disease trajectory and achieve remission [75,86].

Membranous Nephropathy: Rituximab

The therapeutic landscape for MN has been transformed by the MENTOR trial, which randomized 130 patients with primary MN and nephrotic syndrome to rituximab versus cyclosporine [10]. Rituximab demonstrated superior rates of complete or partial remission at 24 months (hazard ratio 3.82, 95% CI 2.42-6.02, p < 0.001), with 60% of rituximab-treated patients achieving remission compared to 20% in the cyclosporine group [10]. Current guidelines recommend rituximab as first-line immunosuppressive therapy for primary MN with nephrotic syndrome [28].

IgAN: Emerging Targeted Therapies

IgAN has historically lacked disease-specific therapies beyond supportive care with RAS inhibition and blood pressure control. However, recent years have witnessed the emergence of multiple targeted therapies addressing different pathophysiologic pathways in IgAN [11].

Budesonide (Nefecon), a targeted-release corticosteroid formulation, demonstrated preservation of kidney function in the NefIgArd trial with a treatment effect on eGFR slope of +3.87 mL/min/1.73 m²/year at two years [11]. Complement inhibitors and endothelin receptor antagonists have also shown efficacy in clinical trials.

Table 3 shows the therapeutic approach for NDKD in diabetic patients.

**Table 3:** Integrated therapeutic approach for NDKD in diabetic patients NDKD: non-diabetic kidney disease, CKD: chronic kidney disease, eGFR: estimated glomerular filtration rate, SGLT2i: sodium-glucose cotransporter-2 inhibitor, RAS: renin-angiotensin system, ACEi: angiotensin-converting enzyme inhibitor, ARB: angiotensin receptor blocker, UACR: urinary albumin-to-creatinine ratio, BP: blood pressure, T2DM: type 2 diabetes mellitus, HbA1c: hemoglobin A1c, CV: cardiovascular, RCT: randomized controlled trial, IV: intravenous, PO: per oral, HR: hazard ratio, GLP-1 RA: glucagon-like peptide-1 receptor agonist.

Therapeutic component	Indication	Target/dosing	Evidence level	Benefits	Monitoring
Universal renoprotection					
SGLT2i	All CKD with eGFR ≥ 20 mL/min/1.73 m²	Empagliflozin 10 mg daily; Dapagliflozin 10 mg daily	Strong (RCTs) [6,19,20]	Slows eGFR decline; reduces CV events	eGFR, volume status
RAS inhibition (ACEi or ARB)	All CKD with albuminuria (UACR >30 mg/g)	Titrate to maximum tolerated dose	Strong (RCTs) [83,84,85]	Reduces proteinuria; slows progression	BP, potassium, creatinine
Blood pressure control	All CKD	Less than 120/80 mmHg if tolerated	Strong (KDIGO 2024) [27]	Slows progression; reduces CV events	Home BP monitoring
Glycemic Control	T2DM	HbA1c target less than 7.0% (individualize)	Strong (guidelines) [28]	Reduces microvascular complications	HbA1c every three months
Disease-specific immunomodulation					
Rituximab	Membranous nephropathy	1000 mg IV at 0 and 2 weeks	Strong (MENTOR RCT) [10]	60% remission rate; HR 3.82 vs cyclosporine	Proteinuria, IgG levels
Budesonide (Nefecon)	IgA nephropathy	16 mg PO daily × 9 months	Moderate (NefIgArd RCT) [11]	Preserves eGFR; reduces proteinuria	eGFR, proteinuria
Adjunctive Therapies					
GLP-1 RA	T2DM + CKD	Per labeling	Moderate (CV trials) [75,77]	Additional kidney + CV protection	Weight, glucose
Finerenone	T2DM + CKD + albuminuria	10-20 mg daily	Strong (RCTs) [21,22]	Additive kidney + CV protection	Potassium, creatinine

Kidney biopsy indications and decision framework

Kidney biopsy remains the reference standard for definitive diagnosis of kidney disease etiology, providing histopathologic information that guides therapeutic decision-making. However, biopsy is an invasive procedure with inherent risks, and appropriate patient selection requires careful consideration of whether biopsy findings would alter management.

Indications for kidney biopsy in diabetic patients

Kidney biopsy should be pursued when the results would substantively change therapeutic management, specifically when NDKD is suspected and potentially treatable with disease-specific immunomodulation. Recommended biopsy indications include atypical features suggesting NDKD (absence of diabetic retinopathy, diabetes duration < 5 years, microscopic hematuria, active urinary sediment, nephrotic syndrome with preserved eGFR), rapid progression (eGFR decline > 5 mL/min/1.73 m²/year, rapidly progressive kidney disease), and systemic features (rash, arthritis, constitutional symptoms, or positive serologies suggesting secondary glomerular disease).

## Conclusions

NDKD is a common, clinically important, and therapeutically distinct cause of renal dysfunction in patients with T2DM, occurring in roughly 30%-50% of diabetic patients who undergo kidney biopsy. Its histopathologic spectrum varies geographically, with IgAN seen most frequently in Asian cohorts and MN and FSGS more commonly reported in Western populations. Several clinical features, including the absence of diabetic retinopathy, shorter diabetes duration, microscopic hematuria, rapid eGFR decline, and nephrotic-range proteinuria with preserved kidney function, can help raise suspicion for NDKD; however, none are accurate enough on their own to confirm or rule it out without a biopsy. Kidney biopsy therefore remains the diagnostic gold standard and should be considered when the results are expected to meaningfully guide treatment decisions. While novel urinary proteomic markers and AI-based diagnostic tools may eventually support non-invasive risk assessment, they currently lack the validation needed to replace histologic evaluation in routine practice.

Therapeutic options have expanded significantly with strong evidence supporting SGLT2i, GLP-1 receptor agonists, and nonsteroidal mineralocorticoid receptor antagonists as kidney-protective therapies across both diabetic and non-diabetic CKD. These broadly renoprotective agents should be combined with disease-specific immunomodulatory treatments when NDKD is present to achieve optimal outcomes. Improving care for kidney disease in diabetic patients requires shifting from narrow, disease-specific frameworks to integrated management strategies that pair universal renoprotection with histology-guided targeted therapy. Clinicians should maintain a strong index of suspicion for NDKD when atypical findings arise, obtain a kidney biopsy when appropriate, and implement comprehensive treatment approaches that address both the underlying glomerular disease and the patient’s broader metabolic and cardiovascular risks.
